# Barriers towards the publication of academic drug trials. Follow-up of trials approved by the Danish Medicines Agency

**DOI:** 10.1371/journal.pone.0172581

**Published:** 2017-05-09

**Authors:** Louise Berendt, Lene Grejs Petersen, Karin Friis Bach, Henrik Enghusen Poulsen, Kim Dalhoff

**Affiliations:** 1 The GCP Unit at Copenhagen University Hospital, Bispebjerg University Hospital, Copenhagen, Denmark; 2 Medicines Development & Clinical Trials, Danish Medicines Agency, Copenhagen, Denmark; 3 Department of Clinical Pharmacology, Bispebjerg University Hospital, Copenhagen, Denmark; 4 Laboratory of Clinical Pharmacology, Rigshospitalet, Copenhagen, Denmark; 5 Faculty of Health Sciences, University of Copenhagen, Copenhagen, Denmark; Royal College of Surgeons in Ireland, IRELAND

## Abstract

**Objective:**

To characterize and quantify barriers towards the publication of academic drug trials.

**Study design:**

We identified academic drug trials approved during a 3-year period (2004–2007) by the Danish Medicines Agency. We conducted a survey among the trial sponsors to describe the rates of initiation, completion, and publication, and the reasons for the failure to reach each of these milestones. Information on size and methodological characteristics of the trials was extracted from the EudraCT database, a prospective register of all approved clinical drug trials submitted to European medicines agencies since 2004.

**Results:**

A total of 181 academic drug trials were eligible for inclusion, 139 of which participated in our survey (response rate: 77%). Follow-up time ranged from 5.1 to 7.9 years. Most trials were randomized controlled trials (73%, 95% CI 65–81%). Initiation and completion rates were 92% (95% CI: 88–97%) and 93% (95% CI: 89–97%) respectively. The publication rate of completed trials was 73% (95% CI: 62–79%). RCTs were published faster than non-RCTs (quartile time to publication 2.9 vs. 3.1 years, p = 0.0412).

**Conclusions:**

Many academic drug trials are left unpublished. Main barriers towards publication were related to the process from completion to publication. Hence, there is much to gain by facilitating the process from analysis to publication. Research institutions and funders should actively influence this process, e.g. by requiring the publication of trial results within a given time after completion.

## Introduction

Data and conclusions from clinical trials are only available to the public if they are published. Nevertheless, it has been estimated [1, 2] that less than half of trials initiated by academic researchers are published. Such underreporting may lead to ethical and scientific problems such as erroneous conclusions due to publication bias, unnecessary repetition of trials, waste of resources, and failure to fulfill the trial subjects’ anticipation of making a contribution to medical knowledge [3–8]. It may also lead to the failure to discontinue the use of a less effective drug or delay the introduction of a more beneficial drug.

All researchers are obligated to make the results of their clinical trials publicly available [9], but limited knowledge is available on the completion and publication of academic drug trials. Ioannidis found that 61% of government-funded HIV trials initiated in 1986–1996 were completed while 55% of completed trials were published [10]. Studies on drug trials generally demonstrate high rates of trial initiation (range: 88–97%[11–15]) and completion (range: 61–95% of initiated trials [10–14]), and lower rates of publication (range: 33–74% of completed trials [10–14, 16]). However, academic drug trials are underrepresented in these studies as the majority of drug trials are conducted by the drug industry. Knowledge on the barriers towards the completion and publication of academic drug trials is needed to improve the reporting of these trials.

It has been indicated that the process from initiation to completion is more critical than the process from approval to initiation [17]. It is unknown whether the initiation and completion rates of academic clinical drug trials are similar as are the reasons for the underreporting of these trials.

The main objective of our study was to characterize and quantify the barriers towards publication of academic clinical drug trials by examining how many trials are initiated, completed, and published. Secondarily, we describe the main reasons for the failure to reach each of these milestones.

## Material

**Fig 1:**
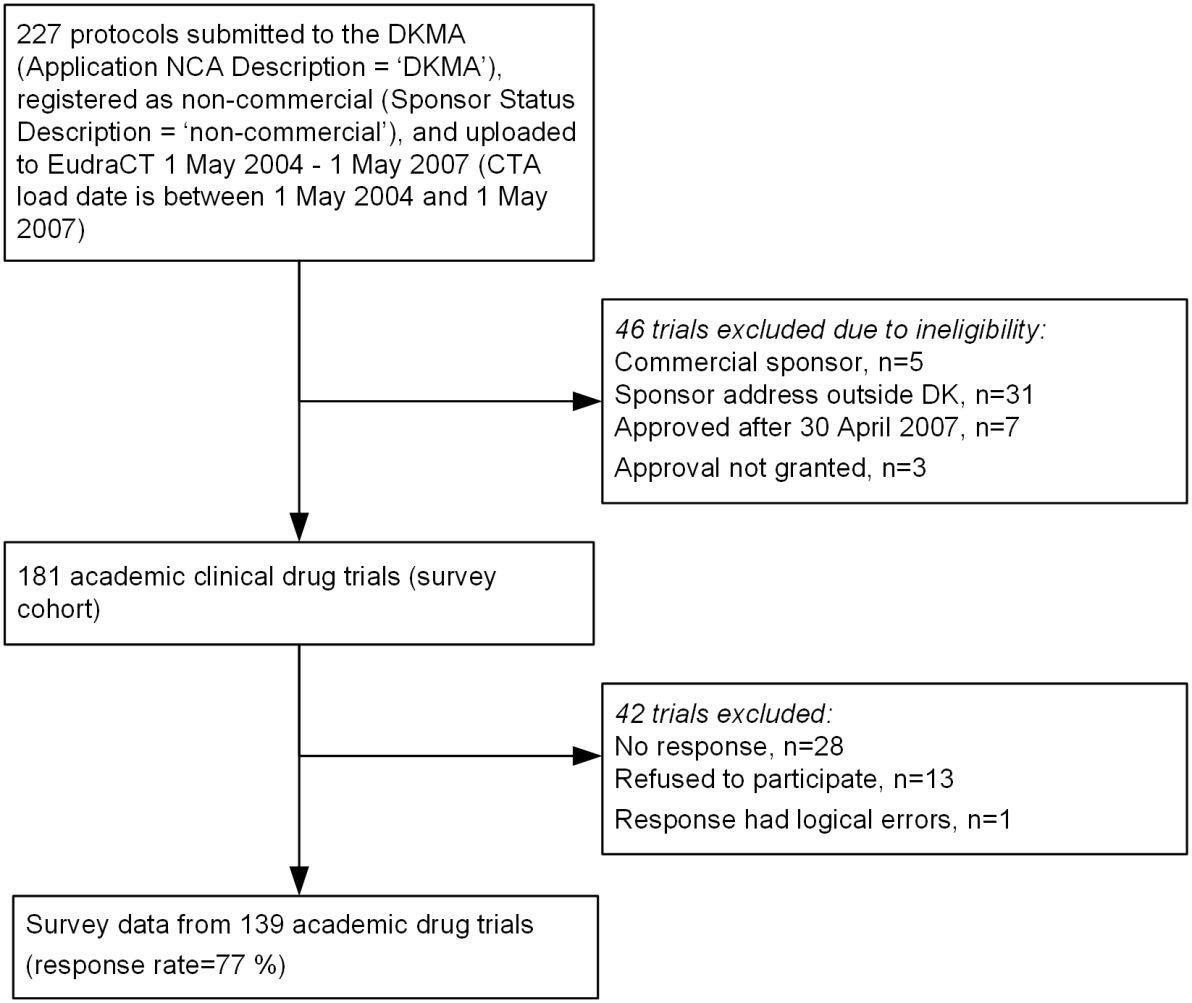
Flow chart.

In May 2012, we identified academic clinical drug trials from the EudraCT database [18]. The database is a prospective registry of all clinical drug trials approved after 1 May 2004 by medicines authorities in the European Community. The European Medicines Agency kindly provided an extract from the EudraCT database based on these inclusion criteria: clinical drug trial applications submitted to the Danish Medicines Agency after 30 April 2004 and uploaded to the database no later than 1 May 2007, sponsor status: non-commercial (see Fig 1). We excluded sponsors that were not resident in Denmark and trial applications approved after 30 April 2007. There were no restrictions regarding funding or phase (I-IV). We defined a trial as academic if trial data and publication rights seemed to belong to a publicly employed researcher and if no company was named on the front page of the protocol. We have previously confirmed the consistency between our definition and the classification as ‘non-commercial’ in the EudraCT database [1, 2]. The dataset contained information on sponsor’s name, sponsor’s contact details, randomization, blinding, design, control groups, and planned number of trial subjects. The contact information for each sponsor was updated with information from a registry of Danish physicians or from searching the internet.

The study was notified to the Danish Data Protection Agency (ID: BBH-2011-23 ACo). Ethical approval was not required according to Danish law.

## Method

The number of trials that were initiated, completed, and published was collected from a survey among sponsors of academic drug trials. We also asked about the statistical significance of the trial results. The characteristics of each trial in terms of randomization, blinding, design, control groups, and planned number of trial subjects were extracted from the EudraCT dataset. We determined the medical specialty from the protocol title, the sponsor, and the sponsor’s affiliation.

The survey was launched 4 June 2012 using an internet-based survey software, Enalyzer^®^. This date was used as the cut-off for the survey. Each sponsor was asked if at least one subject had been included in the trial (initiation milestone), whether the trial had been completed to such an extent that a conclusion could be drawn (completion milestone), and whether the trial was published in a PubMed-, EMBASE-, or Cochrane Library-indexed scientific journal or presented as a poster or similar with an associated indexed abstract (publication milestone). If the trial was completed, the respondent was asked whether the trial conclusion was based on statistical testing, and, if so, whether the outcome was statistically significant. The respondent was also asked to state the reference(s) of associated publication(s). In case of failure to initiate, complete, or publish a trial, the sponsor was asked to grade the influence of a number of suggested reasons (see Table 1) and to fill in additional comments. The selection of reasons were inspired by the findings of Chan [19], Decullier [20], and Weber [21].

**Table 1:** Reasons for non-initiation, non-completion, and non-publication of academic drug trials.

	Degree of influence					
	Not at all influential n (%)	Slightly influential n (%)	Moderately influential n (%)	Very influential n (%)	Do not know n (%)	Missing n
**Reasons for non-initiation (n = 11)**						
Lack of time	5 (50%)	-	3 (30%)	2 (20%)	-	1
Lack of monetary resources	5 (50%)	2 (20%)	1 (10%)	2 (20%)	-	1
Lack of study personnel	5 (50%)	2 (20%)	3 (30%)	-	-	1
Technical difficulties	6 (60%)	1 (10%)	-	2 (20%)	1 (10%)	1
New results from other studies	7 (70%)	1 (10%)	1 (10%)	1 (10%)	-	1
Lack of approval from ethics committee and/or data protection agency	9 (90%)	-	-	1 (10%)	-	1
**Reasons for non-completion (n = 9)**						
Problems with the inclusion of subjects	-	-	2 (25%)	6 (67%)	-	1
Drop out/withdrawal higher than expected	5 (63%)	2 (25%)	1 (13%)	-	-	1
Lack of time	6 (67%)	-	3 (33%)	-	-	-
Lack of monetary resources	6 (67%)	-	2 (22%)	1 (11%)	-	-
Lack of commitment at one or more study sites	6 (75%)	-	1 (13%)	1 (13%)	-	1
Technical difficulties	7 (88%)	-	-	1 (13%)	-	1
Trial ongoing	8 (100%)	-	-	-	-	1
**Reasons for non-publication (n = 20)**						
Data analysis is ongoing	8 (40%)	1 (5%)	2 (10%)	8 (40%)	1 (5%)	-
Manuscript is under preparation	7 (35%)	2 (10%)	4 (20%)	5 (25%)	2 (10%)	-
Lack of time	10 (50%)	2 (10%)	1 (5%)	6 (30%)	1 (5%)	-
The result has been communicated otherwise	12 (60%)	-	2 (10%)	5 (25%)	1 (5%)	-
The result is not interesting	11 (55%)	-	5 (25%)	1 (5%)	3 (15%)	-
The result is not statistically significant	9 (45%)	1 (5%)	4 (20%)	1 (5%)	5 (25%)	-
The results are negative	9 (45%)	1 (5%)	4 (20%)	1 (5%)	5 (25%)	-
Lack of monetary resources	12 (60%)	2 (10%)	3 (15%)	1 (5%)	2 (10%)	-
Lack of study personnel	14 (70%)	-	3 (15%)	1 (5%)	2 (10%)	-
Manuscript has been submitted, but was rejected	17 (85%)	-	-	1 (5%)	2 (10%)	-

Each sponsor was invited to participate in an electronic survey with a personalized email. Reminders were sent after two and three weeks, respectively. Finally, a personalized paper questionnaire was sent to each sponsor’s personal postal address with a stamped return envelope. The second reminder and the paper questionnaire encouraged sponsors of non-initiated, non-completed, and non-published trials to respond.

To roughly estimate the publication rate among trials of survey non-responders we conducted a post-hoc literature search in PubMed. The search was based on the protocol title, investigational drug(s), name of the sponsor, and EudraCT number.

### Analysis

Numbers of initiated, completed, and published trials were listed with cumulative and conditional probabilities. Reasons for not reaching each milestone were tabulated as frequencies and percentages.

We calculated time to publication as the time from the date of approval by the Danish Medicines Agency to the date of acceptance of the first manuscript presenting trial results or, if unavailable, the date of online or paper publication. Time to publication was analyzed using a Cox proportional hazards model, log-rank test, and Kaplan-Meier plots. Trials not published by the launch of the survey (June 4 2012) were censored. Pre-specified covariates were RCT/non-RCT, medical specialty, institution, and type of study (exploratory or confirmatory). We decided post-hoc to include year of approval (i.e. the year in which the clinical trial application was approved by the DHMA) as a covariate and furthermore we did not conduct the analysis with type of study. The association between the statistical significance of trial outcomes and time to publication was analyzed in a 2x2-table with Fischer’s exact test. P-values <0.05 were considered statistically significant.

Information on medical specialty, randomization, blinding, design, control groups, and number of planned subjects were listed with frequencies and percentages. Differences between published and unpublished trials were analyzed with χ^2^ and Fisher’s exact tests.

Data were analyzed in SAS 9.2, GraphPad Prism 6, and GraphPad QuickCalcs. A follow-up interview study among sponsors of non-published trials was planned but not conducted as the number of non-published trials was lower than expected.

## Results

### Identification of trials and conduct of survey

Data from 227 trials were extracted from the EudraCT database, 181 of which fulfilled the eligibility criteria (Fig 1). The survey was sent to the sponsors of 181 Danish academic clinical drug trials. The internet-based survey resulted in 120 responses and 13 refusals including two responses by email. These two sponsors gave informed consent to letting LB fill in the questionnaire during a telephone interview. A further 20 responses and one refusal were obtained from the paper questionnaire. No response was obtained from the remaining 27 sponsors. One trial was excluded due to logical errors (the respondent stated that the trial was not initiated, did not answer the question regarding completion, stated that the trial was published, and cited a publication, which we were not able to find). No logical errors were found in the electronic survey data (the questions were filtered). The final sample consisted of 139 trials (response rate 77%, 139/181). Some respondents had difficulty answering whether statistical significance tests were conducted (2 of 119 completed trials) and whether these tests were significant or inconclusive (3 of 104 completed trials with statistical significance testing), and left these questions blank.

### Characteristics of included trials

Most of the trials were randomized, double blind trials (Table 2). Both randomization and blinding were more prevalent among the published than among the unpublished trials (randomization 80% vs. 62%, *p* = 0.022, blinding 70% vs. 44%, *p* = 0.009). Beside the investigational drug, a non-drug comparator was used in 36% of the trials while an active comparator or a placebo was used in 27% of the trials. Few trials (9%) included more than one type of control group.

**Table 2:** Characteristics of included academic drug trials by publication status.

	All *n* = 139	Published *n* = 84	Unpublished *n* = 50	*p*-value^‡^	Publication status unknown *n* = 5^¤^
**Controlled trials**	110/133 (83%)	71/83 (86%)	37/46 (80%)	0.45	2
Missing	6 (4%)	1 (1%)	4 (8%)	0.06^#^	1
**Controlled trials: Randomization**	92/109 (84%)	64/71 (90%)	26/36 (72%)	0.01	2
Missing	1/110 (1%)	0/71 (0%)	1/37 (3%)	0.35^#^	0
**Controlled trials: Blinding**	67/105 (64%)	50/68 (74%)	16/35 (46%)	0.01	1
Missing	5/110 (5%)	3/71 (4%)	2/37 (5%)	1.00^#^	0
**Controlled trials: Design**					
Parallel groups	30/101 (30%)	17/67 (25%)	12/33 (36%)	0.25	1
Cross-over	26/101 (26%)	24/67 (36%)	2/33 (6%)	<0.01	0
Other design or combination of designs	10/101 (10%)	5/67 (7%)	5/33 (15%)	0.23	0
Missing	44/110 (40%)	25/71 (35%)	18/37 (49%)	0.18	1
**Controlled trials: Control group interventions**					
Comparator other than active or placebo	45/107 (42%)	34/70 (49%)	10/35 (29%)	0.05	1
Active comparator only	19/107 (18%)	13/70 (19%)	6/35 (17%)	0.86	0
No comparator	19/107 (18%)	8/70 (11%)	11/35 (31%)	0.01	0
Placebo only	13/107 (12%)	9/70 (13%)	3/35 (9%)	0.75^#^	1
Active comparator and other comparator	6/107 (6%)	5/70 (7%)	1/35 (3%)	0.66^#^	0
Placebo and other comparator	3/107 (3%)	1/70 (1%)	2/35 (6%)	0.26^#^	0
Active comparator and placebo	2/107 (2%)	0/70 (0%)	2/35 (6%)	0.11^#^	0
Missing	3/110 (3%)	1/71 (1%)	2/37 (5%)	0.27^#^	0
Top five medical specialties*					
1. Clinical oncology	20/139 (14%)	7/84 (8%)	13/50 (26%)	0.01	0
2. Neurology	21/139 (15%)	18/84 (21%)	3/50 (6%)	0.02	0
3. Anesthesiology	15/139 (11%)	12/84 (14%)	2/50 (4%)	0.06	1
4. Gynecology/obstetrics	12/139 (9%)	4/84 (5%)	7/50 (14%)	0.06	1
5. Endocrinology	7/139 (5%)	4/84 (5%)	3/50 (6%)	1.00	0
**Median planned sample size**	50	49	58	-	30
10^th^ and 90^th^ percentiles	16–200	12–150	20–238	-	20–108

^‡^χ^2^ test.

^#^Fisher’s exact test due to expected cell counts <5.

^¤^ Information was missing (n = 3) or conflicting (n = 2).

*Four trials had two medical specialties

A total of 21 medical specialties were represented in the cohort. The five most frequent specialties were clinical oncology (n = 21), neurology (n = 21), anesthesiology (n = 15), gynecology/obstetrics (n = 12), and endocrinology (n = 7), Table 2.

### Initiation, completion, and publication of academic drug trials

By the end of follow-up on 4 June 2012, 84 trials were published, 50 were not published, and 5 had unknown publication status (Table 3). Overall, 61% (95% CI: 53–69%) of approved trials were published. While 92% (95% CI: 88–97%) of the approved trials were initiated and 93% (95% CI: 89–97%) of the initiated trials were completed, only 71% (95% CI: 62–79%) of the completed trials were published. Sixteen of the 30 unpublished trials had been disseminated otherwise, e.g. presented at a scientific meeting or conference. Rates of initiation, completion, and publication were similar across the period under study (Table 4).

**Table 3:** Conditional probability of initiation, completion, and publication of approved academic drug trials (success = the reaching of a given milestone, failure = the failure to reach a given milestone).

					Conditional probability		Cumulative probability
Milestone	n(failure)	n(success)	n(unknown)	N	of failure	of success	of success
Initiation	11	128	0	139	0.08	0.92	0.92
Completion	9	119	0	128	0.07	0.93	0.86
Publication	30	84	5^#^	119	0.25	0.71	0.61

^#^ Information was missing (n = 3) or conflicting (n = 2).

**Table 4:** Number and proportion of initiated, completed, and published academic drug trials by year of approval of clinical trial application.

	All trials	Initiated trials	Completed trial	Published completed trials	Unpublished completed trials	Publication status unknown	Median time from approval to publication
Year of approval	*n* = 139	*n* = 128	*n* = 119	*n* = 84	*n* = 30	*n* = 5*	Years (95% CI)
2004^#^	11	10 (91%)	9 (90%)	7 (78%)	1 (11%)	1	5.1 (1.3–5.4)
2005	54	49 (91%)	44 (90%)	27 (61%)	15 (34%)	2	5.2 (4.1–6.8)
2006	61	57 (93%)	54 (95%)	41 (76%)	11 (20%)	2	3.7 (3.0–5.0)
2007^‡^	13	12 (92%)	12 (100%)	9 (75%)	3 (25%)	-	4.2 (1.8–5.3)

^#^Trials submitted after 30 Apr 2004 and approved by 31 Dec 2004.

^‡^Trials approved 1 Jan 2007 to 30 Apr 2007.

*Information on publication was missing (n = 3) or conflicting (n = 2).

**Fig 2:**
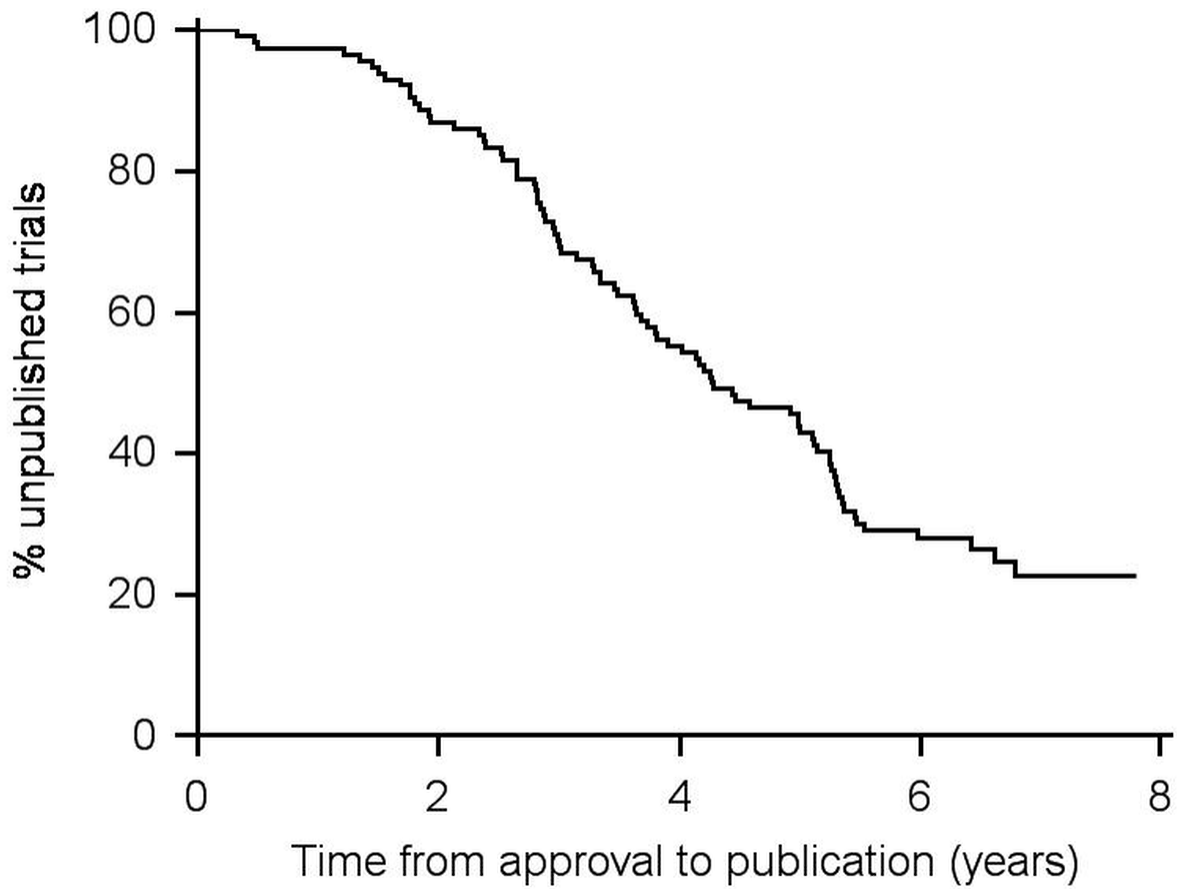
Time from approval to publication of completed academic drug trials. (n = 114).

**Fig 3:**
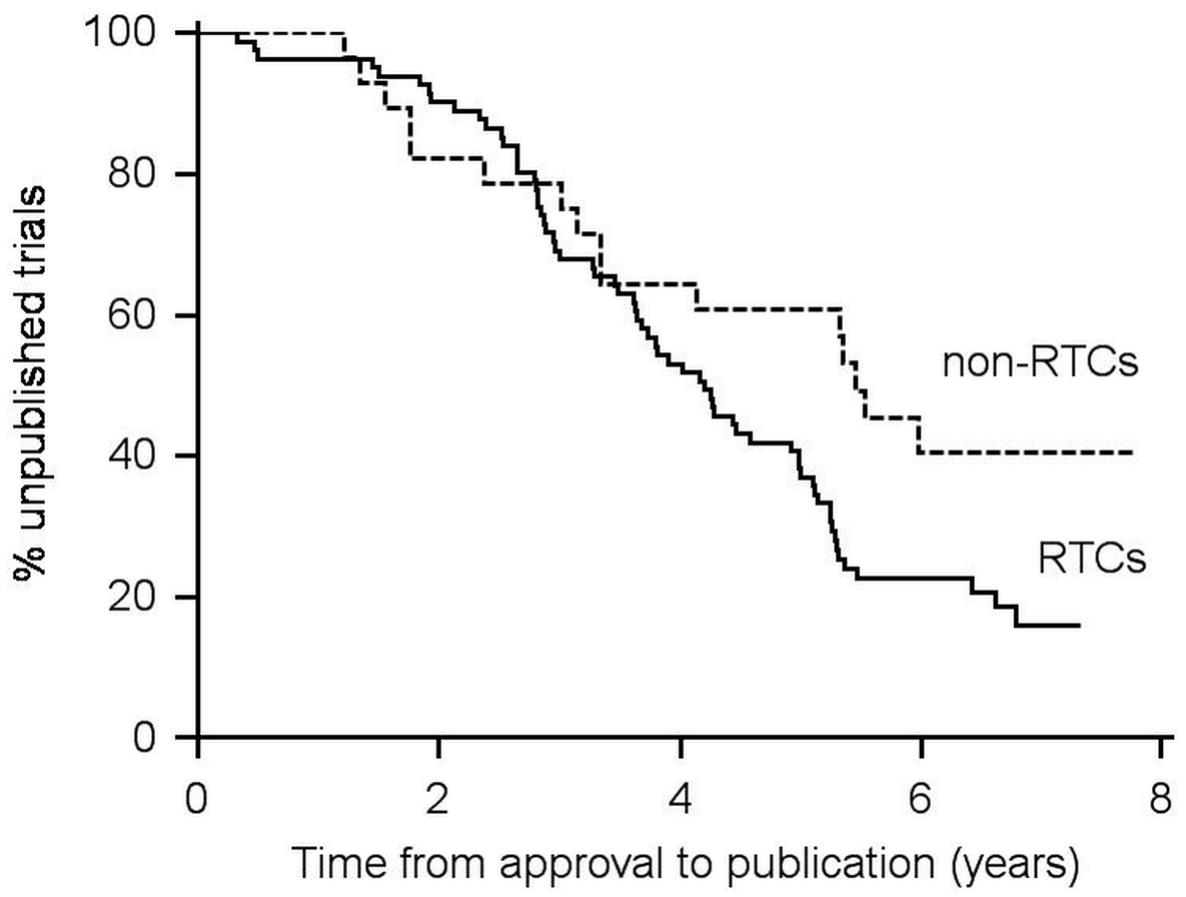
Time from approval to publication of completed academic drug randomized controlled trials (RCTs) and non-randomized controlled trials (non-RCTs).

Kaplan-Meier plots of time from approval to publication are shown in Fig 2. Median time from approval to publication was 5.2 years (95% CI: 4.3–5.5). RCTs were published faster than non-RCTs (p = 0.041, log-rank test). The median time from approval to publication of RCTs was 4.2 years (95% CI: 3.7–5.1), but could not be computed for non-RCTs due to the low publication rate. Instead we compared the quartile time from approval to publication, which was 2.9 years (95% CI: 2.5–3.3) and 3.1 years (95% CI: 1.3–5.3) for RCTs and non-RCTs respectively (Fig 3).

Median time from approval to publication was not associated with the year of approval (p = 0.07, log-rank test). However, we observed a tendency towards a shorter median time to publication among trials approved in 2006 and 2007 than trials approved in 2004 and 2005 (Table 4).

According to the sponsors, statistically significant results were obtained in 69 of 91 (72%) completed trials with known publication status and a conclusion based on the statistical testing of a hypothesis. The publication rate was 84% among trials with a statistically significant result compared to 86% among trials with a statistically insignificant result (OR 0.8; 95% CI: 0.2–3.3, p = 1.00).

Reasons for non-initiation, non-completion, and non-publication are listed in Table 1.

#### Reasons for non-initiation

Eleven of the 139 approved trials were not initiated. Lack of time was the most frequently stated reason (n = 5). Lack of study personnel and monetary resources were stated as influential by five sponsors each. One sponsor stated that the trial was abandoned because the regional GCP unit refused to monitor the trial due to ethical issues. The sponsor also stated that the trial was approved by both the ethics committee and the Danish Medicines Agency. One sponsor stated that the trial was not initiated due to personal reasons.

#### Reasons for non-completion

Nine studies were initiated but not completed. The most frequent reason for this was problems with the inclusion of trial subjects. This was graded moderately or very influential by the sponsors of eight of the nine non-completed trials. One study was stopped prematurely due to side effects. One sponsor stated that the ethics committee’s demand for the listing of cancer as a potential side effect to the investigational drug in the patient information leaflet made it almost impossible to find patients willing to participate. Neither of the sponsors stated ‘trial ongoing’ as influential of non-completion.

#### Reasons for non-publication

The analysis of the reasons for non-publication was based on the 20 unpublished trials that had neither been accepted for journal publication nor presented at a scientific conference or similar with an associated indexed abstract. The most frequently given factors influential of non-publication was ongoing data analysis (11/20), manuscript under preparation (11/20) and lack of time (9/20). Eight of the eleven responses stating ongoing data analysis as influential of non-publication had chosen ‘highly influential’. Similarly, ‘highly influential’ was chosen by five of the 11 respondents deeming the ongoing manuscript preparation as influential. One respondent explained that the results of the trial were indeed interesting, but as other parts of the respondent’s job were now more demanding than when planning the trial, the publication process had slowed down. Lack of time was the third most frequent reason deemed influential of non-publication with six of the nine respondents grading lack of time as highly influential. One respondent wrote that the trial was still including subjects.

The literature search for publications from the 41 non-responders yielded a publication rate of 41% (17/41).

## Discussion

We found an overall publication rate of 61% (71% of completed trials) by surveying sponsors of approved academic drug trials. Almost all approved trials were initiated and completed. Hence, the process from completion to publication seems more problematic than does the process from approval to completion of a trial. Main reasons for non-publication of completed trials indicated that data analysis and manuscript preparation were often still going on. Reasons for non-initiation were mainly related to lack of resources while non-completion was primarily related to problems with the inclusion of trial subjects.

The initiation and completion rates observed in our study (92% and 93%, respectively) seem comparable to those previously reported from cohorts of clinical drug trials [10–14, 16]. High initiation and completion rates may indicate that planned trials are feasible and only few barriers not overcome during the conduct of academic drug trials. This would support our previous results showing that academic researchers are able to conduct drug trials under the same comprehensive regulation as the drug industry [2].

We found a publication rate of 61% of approved trials (71% of completed trials), which seems high compared to previous studies (range: 33–74% of completed trials [10–14, 16]) and comparable to a recent study by van den Bogert (clinical drug RCTs approved by Dutch Independent Review Boards in 2007)[15]. The difference to previous studies may be attributed to several factors: Firstly, the publication rate may have increased over time. Our cohort of trials approved in 2004–2007 comprise more recent trials than those studied by Suñé[11] (drug trials approved in 1997–2004), Hole [16] (drug trials approved in 2000), Berendt [1] (academic drug trials submitted in 1993–2005 and 1999–2003), Decullier [12] (drug trials approved in 1994), Ioannidis [10] (non-commercial HIV drug trials initiated in 1986–1996), von Elm [13] (drug RCTs approved in 1989–1998), and Bardy [14] (drug trials approved in 1987). Since then the framework for the conduct of clinical drug trials has changed remarkably. These changes include the demand by the International Committee of Journal Editors (ICMJE) to prospectively register clinical trials initiated after 1 July 2005 in a publicly available database [22]. They also include the obligation for all drug trials conducted in the European Community to adhere to the principles of Good Clinical Practice (ICH-GCP)[23]. Thirdly, the publication rate of academic drug trials may be higher than that of commercial drug trials. This is supported by data from von Elm showing that non-commercial funding was associated with an increased probability of publication [13], but contrasts the publication rates reported by Hole et al [16] of 73% and 75% among ‘sponsored’ and ‘non-sponsored’ completed trials respectively. Fourthly, sponsors of published trials may have been more likely to respond to our survey than the sponsors of non-published trials. This would inflate the observed publication rate. However, combination of the observed publication rate (61%, 84/139) with that of the PubMed search for publications of the trials of non-responders (41%, 17/41) yielded a publication rate of 56% (101/180). Although this is a rough estimate, it suggests the inflation of the observed publication rate to be of limited size. We found no indication that the risk of bias in our study is different from previous studies of drug trials. The response rate of 77% (139/181) is comparable to the response rates obtained in previous studies (median: 100%, range: 67–100%).

The observed difference in the time from approval to publication of RCTs and non-RCTs may reflect underlying differences between the two groups rather than the design itself. Oncology trials are typically of long duration. They constituted 34% of non-RCTs (16/47), but only 4% of RCTs (4/92). This bias would probably have been minimized had we used the time from completion to publication instead of the time from approval to publication. However, the time of approval is better documented than the time of completion, which may be subject to recall bias.

We observed a tendency towards a reduction in the time from approval to publication from 2004 to 2007. A larger sample is needed to evaluate whether this reflects an actual shortening. Other factors than the efficiency of researchers may have influenced the observed time to publication, e.g. a shift towards more short-term trials and fewer long-term trials. Because we measured the time from approval rather than completion we could not differentiate the effect of such changes from a change in the time used for the processing and publishing of trial results.

A Cochrane review found that trials with positive findings are published faster and more frequently than other trials [3]. We did not observe an association between publication and the presence of a statistically significant outcome when analyzing our data in a 2x2 table. Neither did Callaham [24], but Schmucker et al. found an association between publication and direction of study findings in a large-scale meta-analysis of observational and interventional studies [25]. The difference may be due to the limited sample size of our study, or to underlying differences in our narrow cohort compared to the broad cohort analyzed by Schmucker. In our study, data on the statistical significance of trial results originated from the survey respondents and may hence be associated with a degree of uncertainty.

Song [26] states that publication bias only arises when published studies are not representative of all studies conducted. But from an ethical point of view random non-publication is problematic as well. The failure to publish a conducted study means that more trials will need to be conducted, and more patients will be at risk of experiencing side effects or lack of treatment effect. Furthermore, the completion of a trial is associated with a large build-up of knowledge and the failure to publish the results may lead to a greater loss of knowledge and waste of resources than the failure to initiate or complete a trial.

According to the trial sponsors, the main reasons for non-initiation were lack of resources (time, money, personnel) while non-completion were mainly due to problems with the inclusion of trial subjects. Main reasons for non-publication were ongoing data analysis or the preparation of a manuscript. This is, however, no guarantee of future publication. In line with previous findings [19, 27–29] few respondents (only one) stated the rejection of a manuscript as influential on the failure to publish a trial. Decullier [12] also reported inclusion problems as the main reason for non-completion, but other reasons for non-initiation and non-publication, e.g. confidentiality issues. The differences may be due to different composition of the two cohorts as the study by Decullier primarily included commercial trials. In addition, numbers of non-initiated, non-completed, and non-published trials are relatively small in both studies.

Half of the unpublished trials had been disseminated at a conference or similar with an associated indexed abstract. Thus, non-publication was not synonymous to non-dissemination, but only the indexing in bibliographic databases facilitates the systematic identification of trial results by other researchers, reviewers, etc.

### Strengths and limitations

Our study is the first to study the completion and publication of academic drug trials that were conducted under the principles of Good Clinical Practice (GCP). A major strength is that we studied the process in an inception cohort as described by Ioannidis [10] and Song [26] by including all Danish academic clinical drug trials prospectively registered in the EudraCT database. We believe that the vast majority of drug trials conducted in Denmark are registered in the database. Large numbers of missing values—especially on design and blinding—may limit the validity of some of our results. A check for consistency in the information on randomization and the use of a control group showed that the information was consistent in 127 of 130 trials. Three trials had conflicting information as they were registered as both uncontrolled and randomized. Moreover, the proportion of controlled trials (83%), RCTs (73%) and blinded trials (62%) in our study cohort seems comparable to our earlier findings of 79% controlled trials, 68% RCTs and 47% blinded trials in a sample of Danish academic drug trials approved from 1993 to 2005 [1].

Our results reflect the framework for clinical trials as it was when the trials in our cohort were conducted and reported. Since then, new initiatives to improve transparency, accessibility and publication rates have been introduced. In 2011, selected information on clinical drug trials approved by European medicines authorities was made publicly available [30]. Furthermore, sponsors of clinical drug trials in the European Community are now obliged to post results-related information to the EudraCT database following end of trial [31]. Both protocol-related and results-related data are publicly available from the EU Clinical Trials Register [30]. This will hopefully improve access to unpublished results. A major limitation is the lack of indexing in bibliographic databases. Therefore, the data will not be easily available when searching for literature.

### Conclusion

The observed publication rate was higher than expected, but many academic drug trials are still left unpublished. Main barriers towards publication were related to the process from completion to publication, e.g. data analysis and manuscript preparation still going on 5 to 8 years after approval of a trial. This suggests that much may be gained by facilitating the process from analysis to publication. Besides regulatory initiatives, research institutions and funders should also actively influence this process, e.g. by requiring the publication of trial results within a given time after completion.

## Supporting information

S1 File (XLS)
